# Protamine neutralizes chondroitin sulfate proteoglycan-mediated inhibition of oligodendrocyte differentiation

**DOI:** 10.1371/journal.pone.0189164

**Published:** 2017-12-07

**Authors:** Kazuya Kuboyama, Naomi Tanga, Ryoko Suzuki, Akihiro Fujikawa, Masaharu Noda

**Affiliations:** 1 Division of Molecular Neurobiology, National Institute for Basic Biology (NIBB), Okazaki, Aichi, Japan; 2 School of Life Sciences, The Graduate University for Advanced Studies (SOKENDAI), Okazaki, Aichi, Japan; Hannover Medical School, GERMANY

## Abstract

Chondroitin sulfate proteoglycans (CSPGs), which are enriched in demyelinating plaques in neurodegenerative diseases, such as multiple sclerosis (MS), impair remyelination by inhibiting the migration and differentiation of oligodendrocyte precursor cells (OPCs) in the central nervous system (CNS). We herein show that protamine (PRM, also known as a heparin antagonist) effectively neutralizes the inhibitory activities of CSPGs, thereby enhancing OPC differentiation and (re)myelination in mice. Cell-based assays using mouse OPC-like OL1 cells revealed that the PRM treatment exerted masking effects on extracellular CSPGs and improved oligodendrocyte differentiation on inhibitory CSPG-coated substrates. PRM also bound to the extracellular region of protein tyrosine phosphatase receptor type Z (PTPRZ), a membrane-spanning CSPG predominantly expressed in OPCs, and functioned as a ligand mimetic of PTPRZ, thereby suppressing its negative regulatory activity on oligodendrocyte differentiation. In primary cultures, the differentiation of OPCs from wild-type and *Ptprz*-deficient mice was equally enhanced by PRM. Moreover, the intranasal administration of PRM accelerated myelination in the developing mouse brain, and its intracerebroventricular administration stimulated remyelination after cuprizone-induced demyelination. These results indicate that PRM has CSPG-neutralizing activity which promotes oligodendrocyte differentiation under developmental and morbid conditions.

## Introduction

Myelination is an essential feature of the vertebrate nervous system that provides electrical insulation to axons in order to facilitate the transmission of nerve impulses, and also functions to maintain long-term axonal integrity [1–3]. Deficiencies in myelination in the central nervous system (CNS) lead to neurological disorders during development or in adulthood in diseases or following injury [4–7]. Multiple sclerosis (MS) is a chronic autoimmune and neurodegenerative disease that is characterized by immune cell infiltration, demyelination, and neuroaxonal damage [8]. Immunomodulatory therapy for MS with interferon β and glatiramer acetate is the most common treatment to reduce the frequency of relapses and slow the progression of its associated disabilities [8]. There are currently 15 disease-modifying medications approved by the Food and Drug Administration (FDA) for use in relapsing forms of MS; the most up-to-date information on-line is available at www.nationalMSsociety.org. Among these, ocrelizumab (anti-CD20) is also the first medication recently approved for primary progressive MS; mitoxantrone is also approved for secondary progressive MS [9]. Although these therapies effectively control inflammation, they dose not promote the regeneration of new myelin sheaths [9].

Potential causes of remyelination failure in MS are manifold and include the presence of extrinsic inhibitors in lesions, insufficient pro-regenerative factors, and also an impaired intrinsic capacity in oligodendrocyte lineage cells [10]. The rationale for the concept of remyelination therapy in MS has been demonstrated by improving clinical outcomes in several mouse demyelination models [10]. LINGO-1 (leucine-rich repeat and immunoglobulin domain-containing Nogo receptor-interacting protein-1), a negative regulator of FYN kinase, is the first molecular target in developing remyelination therapies. Antagonism of LINGO-1 or its pathway promotes remyelination in mice [11], and anti-LINGO-1 monoclonal antibody BIIB033 is being tested in clinical trials [12].

Chondroitin sulfate proteoglycans (CSPGs) are a family of large molecules consisting of a core protein and repeating disaccharide units (glycosaminoglycans, GAGs). CSPGs function as guidance cues or signaling molecules during development, and maintain the structural integrity of specialized structures such as perineuronal nets and nodes of Ranvier [13, 14]. CSPGs are also known to be enriched in glial scars responsible for poor axonal regeneration and failed functional recovery after traumatic CNS lesions [14, 15]. The glial scar functions as a barrier to limit spreading of tissue destruction after CNS damages [16]. Reactive astrogliosis is associated with glial scar formation after CNS insults [17]. A very recent study revealed molecular details in the astrogliosis after mouse spinal cord injury [18], showing scar-forming astrocytes-specific increases in the expression of two CSPGs, aggrecan and phosphacan: Phosphacan is also called PTPRZ-S referring to the secretory splicing isoform of protein tyrosine phosphatase receptor type Z (PTPRZ) [19, 20].

**Fig 1:**
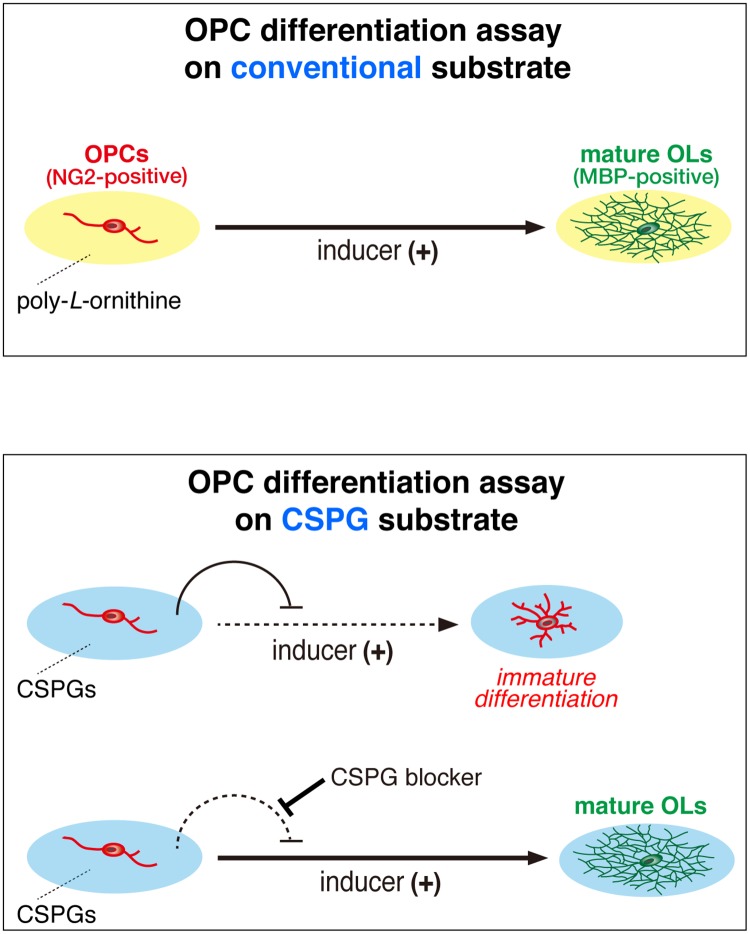
Schematic representation of cell-based assays for oligodendrocyte differentiation activators. Many small molecules with the ability to enhance OPC differentiation and remyelination *in vivo* have been reported to date: ARA-014418 (glycogen synthase kinase 3 (GSK3) β inhibitor) [23], 9-*cis*-retinoic acid (retinoid X receptor agonist) [24], rolipram (phosphodiesterase (PDE) 4 inhibitor) [25], benztropin (M1/M3 muscarinic receptor agonist) [26], clemastine (antihistamine and anticholinergic) [27], quetiapine (antipsychotic drug) [28], solifenacin (M3 inhibitor) [29], clobetasol (synthetic adrenocortical hormone), miconazole (antifungal agent) [30], tamoxifen (estrogen receptor modulator for ERα, ERβ, and GPR30) [31], U-50488 (kappa-opioid receptor agonist) [32], XAV939 (tankyrase inhibitor) [33], and VP3.15 (PDE7 and GSK3 dual inhibitor) [34]. However, most of these do not have the potential to overcome the inhibitory activities of CSPGs. We used OL1 cells cultured on aggrecan-coated substrates as a model of CSPG deposition on demyelinated plaques, and screened compounds that overcome inhibitory activity against differentiation of oligodendrocyte precursor cells (OPCs) to oligodendrocytes (OLs).

In MS lesions, CSPGs including aggrecan, versican, and neurocan accumulate as constituents of demyelinating plaques, thereby inhibiting the differentiation of oligodendrocyte precursor cells (OPCs) and remyelination [15, 16, 21, 22]. Many small molecules with the ability to enhance OPC differentiation and remyelination *in vivo* have been reported to date (for each drug, see Ref [23–34], and summarized in the legend of Fig 1). However, most of these do not have the potential to overcome the inhibitory activities of CSPGs. A very recent study showed that the effects of benztropin, clemastine, quetiapine, and clobetasol on OPC differentiation were significantly suppressed on CSPG-coated substrates [35]. On the other hand, several approaches to neutralize inhibitory CSPGs after injury have been reported, such as the enzymatic digestion of CSPGs with bacterial chondroitinase ABC [16, 21], and the inhibition of CS chain polymerization enzymes with xyloside [15] or fluorosamine [35], which are CSPG synthesis inhibitors.

Remyelination occurs endogenously by the recruitment of OPCs to injured sites and their differentiation to mature oligodendrocytes in order to remyelinate damaged axons. However, most lesions in MS patients fail to completely remyelinate, and chronically demyelinated axons are prone to irreversible damage and loss. This inability to remyelinate is not due to the absence of OPCs in the adult CNS because OPCs are present in elderly patients after decades of disease [36]. These findings have focused our efforts to develop therapeutic strategies that promote remyelination.

We previously established a pure OPC-like population (OL1) of cells from the *p53*-deficient newborn mouse brain [37]. Using OL1 cells as a model system, we herein developed a method to screen neutralizing or masking agents for inhibitory CSPGs (see Fig 1). We found that protamine (PRM) effectively promoted developmental myelination in mouse pups and remyelination in a cuprizone-induced demyelination model. This study thus suggested the possibility that development of CSPG-neutralizing agents is a promising strategy for treating the developmental retardation of myelination and demyelinating diseases.

## Results

### Aggrecan inhibited OL1 differentiation to oligodendrocytes: A cell-culture assay model in demyelinating plaques

**Fig 2:**
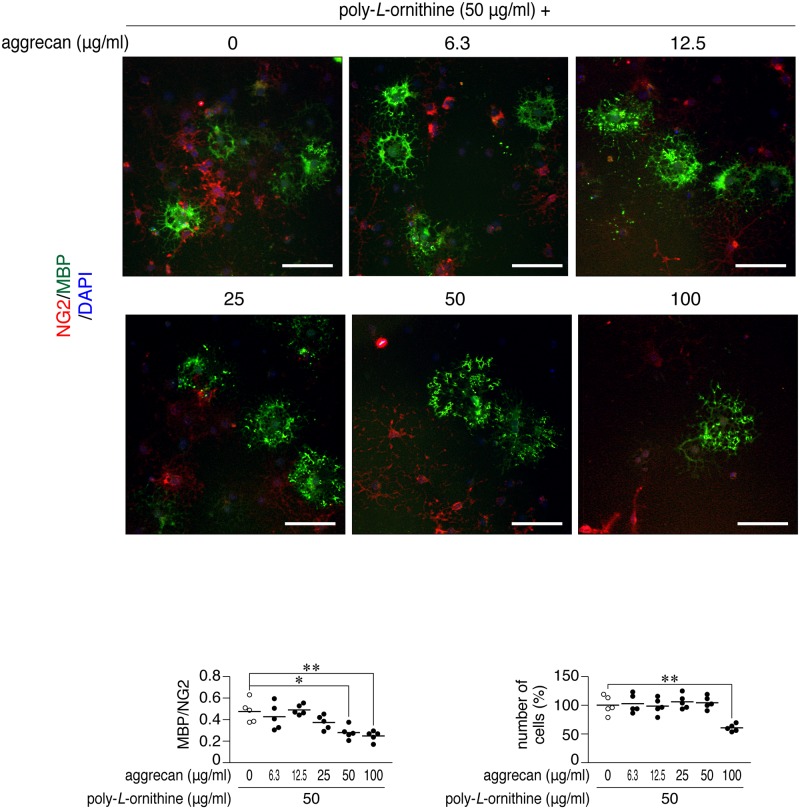
Cell-based phenotypic assays using OL1 cells. Double-immunofluorescence labeling of OL1 cells (upper). OL1 cells were cultured with differentiation medium containing thyroid hormones on dishes coated with poly-*L*-ornithine with/without aggrecan at the indicated concentrations. After 10 days, cells were fixed with formalin and stained with anti-NG2 proteoglycan (OPCs; *red*) and anti-MBP (mature oligodendrocytes; *green*) antibodies, in conjunction with the 4′,6-diamidino-2-phenylindole (DAPI) labeling of nuclei (*blue*). Scale bars, 100 μm. Scatter plots show the ratio of MBP-positive cells to NG2-positive cells (lower left), and total DAPI-positive cell numbers relative to the average of the vehicle control (lower right). Each circle corresponds to an independent cell culture (*n* = 5 each). *, *p* < 0.05 and **, *p* < 0.01, significant difference between the indicated groups (analysis of variance with Bonferroni’s *post-hoc* tests).

As an *in vitro* model of demyelinating plaques, we coated culture dishes with increasing concentrations of aggrecan together with a fixed concentration of poly-*L*-ornithine (Fig 1). Poly-*L*-ornithine is commonly used as a coating substrate to facilitate OPC adhesion and growth. We herein employed aggrecan because it is produced by scar-forming astrocytes [18] and known as a major CSPG component in demyelinating plaques in the CNS [22]. We seeded OL1 cells [37] on these dishes, and cultured them for 10 days under differentiation conditions with thyroid hormones (TH). The differentiation of immature OPC-like OL1 cells into mature oligodendrocytes was evaluated by the ratio of cell numbers of myelin basic protein (MBP)-positive mature cells to neural glial antigen-2 chondroitin sulfate proteoglycan (NG2)-positive immature cells. The aggrecan coating inhibited the differentiation of OL1 cells in a dose-dependent manner, and an apparent reduction in cell adhesion was observed with the coating at a higher concentration of 100 μg/ml (Fig 2). The aggrecan-induced response of OL1 cells was similar to that of OPCs reported previously by others [35, 38, 39].

**Fig 3:**
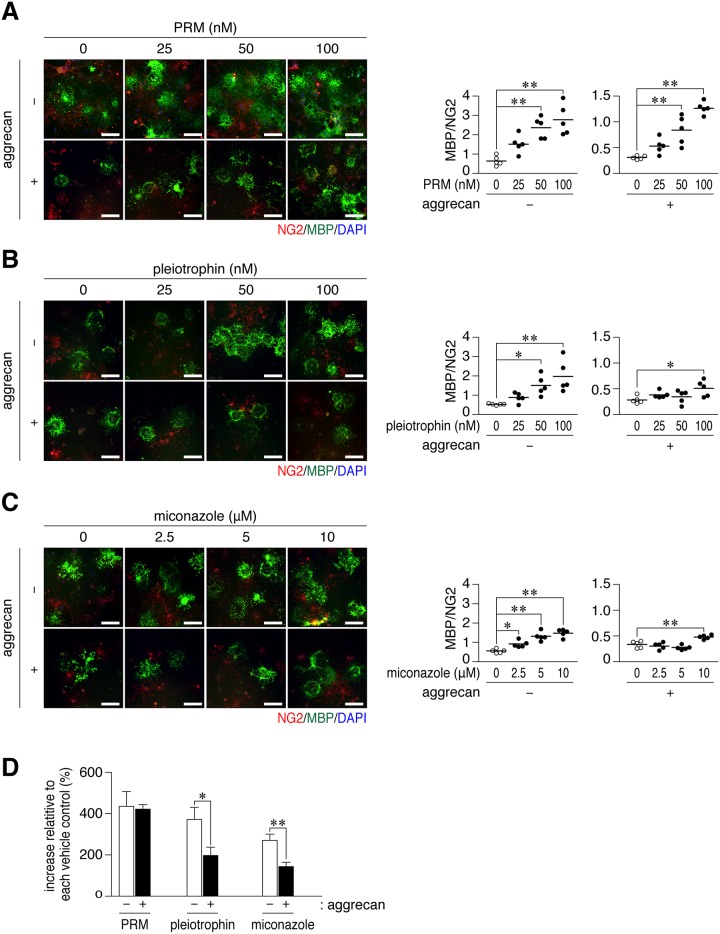
Protamine enhanced the differentiation of OL1 cells on aggrecan-coated dishes. (**A-C**) Anti-NG2 and anti-MBP staining of OL1 cells. OL1 cells were cultured for 10 days on dishes coated with 50 μg/ml poly-*L*-ornithine (-), or a combination of 50 μg/ml poly-*L*-ornithine and 50 μg/ml aggrecan (+). Protamine (PRM, A), pleiotrophin (B), or miconazole (C) was added to differentiation medium at the indicated concentrations. Scale bars, 100 μm. The plots on the right side show the ratio of MBP-positive cells to NG2-positive cells in five independent cell cultures. *, *p* < 0.05 and **, *p* < 0.01, significant difference between the indicated groups (analysis of variance with Bonferroni’s *post-hoc* tests). (**D**) The percentage of the ratio of MBP to NG2 in 100 nM PRM, 100 nM pleiotrophin, or 10 μM miconazole to each vehicle is shown, demonstrating a small effect with pleiotrophin and miconazole on aggrean-coated substrates. Data were the mean with S.E. (*error bars*) from five independent experiments. *, *p* < 0.05 and **, *p* < 0.01, significant difference between the indicated groups (Student’s *t*-tests).

We searched for agents that block (or neutralize) the inhibitory effects of CSPGs on OL1 differentiation. OL1 cells were cultured with a candidate compound individually on a culture plate coated with aggrecan (50 μg/ml) and poly-*L*-ornithine (50 μg/ml) under TH-stimulated conditions. Among the chemical compounds and peptidic biomolecules tested, we found that protamine (PRM) exhibited the most potent activity to differentiate OL1 cells (upper panels, Fig 3A). PRM is a natural arginine-rich cationic polypeptide usually isolated from salmon sperm. Other candidates were ultimately dropped because of their relatively low activity or poor reproducibility. PRM has long been used as a drug or antidote to neutralize the anticoagulant heparin [40], while its promoting effects on CNS myelination and therapeutic potential in demyelinating diseases have not been reported. Moreover, to the best of our knowledge, there have been no studies on PRM binding to aggrecan. In the present study, we found that PRM efficiently bound to aggrecan-coated substrates by an enzyme-linked-immunosorbent assay (S1 Fig).

### PRM neutralized the inhibitory activity of aggrecan on OL1 cell differentiation

PRM enhanced TH-induced differentiation in OL1 cells cultured on non-aggrecan-coated dishes (Fig 3A, upper panels) and aggrecan-coated dishes (Fig 3A, lower panels). However, it did not induce cell differentiation under non-differentiation conditions without TH (S2 Fig), suggesting that PRM has a function to eliminate the blockage of cell differentiation, but not to directly promote cell differentiation. The ratio of MBP-positive cells to NG2-positive cells was increased by PRM from 0 nM to 100 nM to a similar extent between aggrecan-coated dishes and non-coated dishes (Fig 3D, PRM). PRM directly bound to OL1 cells (S3 Fig), suggesting that PRM bound to specific cellular receptors.

**Fig 4:**
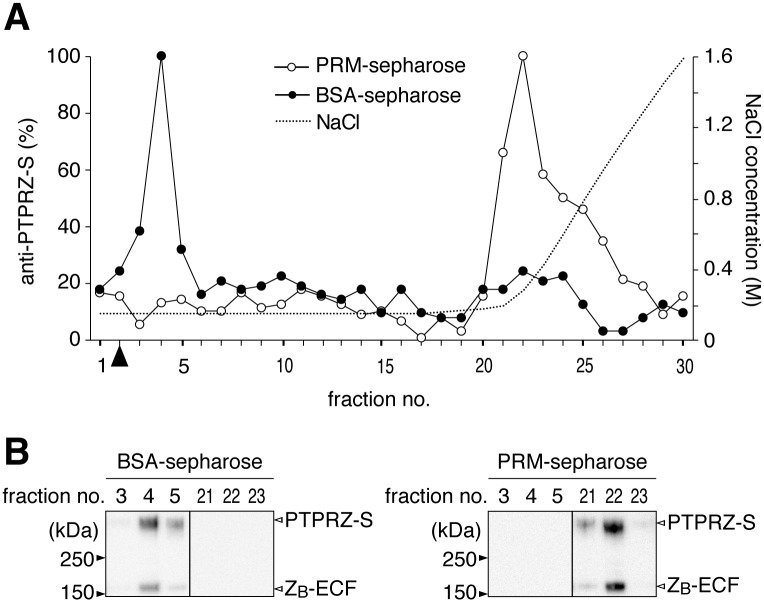
Extracellular portions of PTPRZ bearing CS were efficiently captured on PRM-immobilized resins. (**A**) Elution profiles. Phosphate buffer extracts of mouse brains were applied to a Sepharose column immobilized with PRM or control BSA, and separated using 0.15 to 2.0 M NaCl gradient elution in 10 mM phosphate buffer, pH 7.3. Aliquots of the separated fractions were coated on microtiter wells, and the content of PTPRZ proteins was assessed using anti-PTPRZ-S. Arrowhead, the void volume. (**B**) Western blotting of eluted fractions. Relevant fractions separated by BSA-sepharose (left) and PRM-sepharose (right) were analyzed by Western blotting using anti-PTPRZ-S (a rabbit polyclonal antibody that recognizes an extracellular epitope of PTPRZ). Full-length blots are presented in S8 Fig.

PTPRZ proteins in OPCs is heavily modified with CS chains [37, 41], and functions to suppress their cellular differentiation [41]. Pleiotrophin, a heparin-binding growth factor, enhances TH-induced oligodendrocyte differentiation by acting as an inhibitory PTPRZ ligand [20, 37, 41]. The CS moiety of PTPRZ is essential for achieving the high-affinity binding of pleiotrophin [42]. CS-modified PTPRZ proteins efficiently bound to PRM-immobilized resins, and were released by an increase in the ionic strength of the mobile phase solution, suggesting an electrostatic interaction between cationic PRM and anionic CS chains (Fig 4).

**Fig 5:**
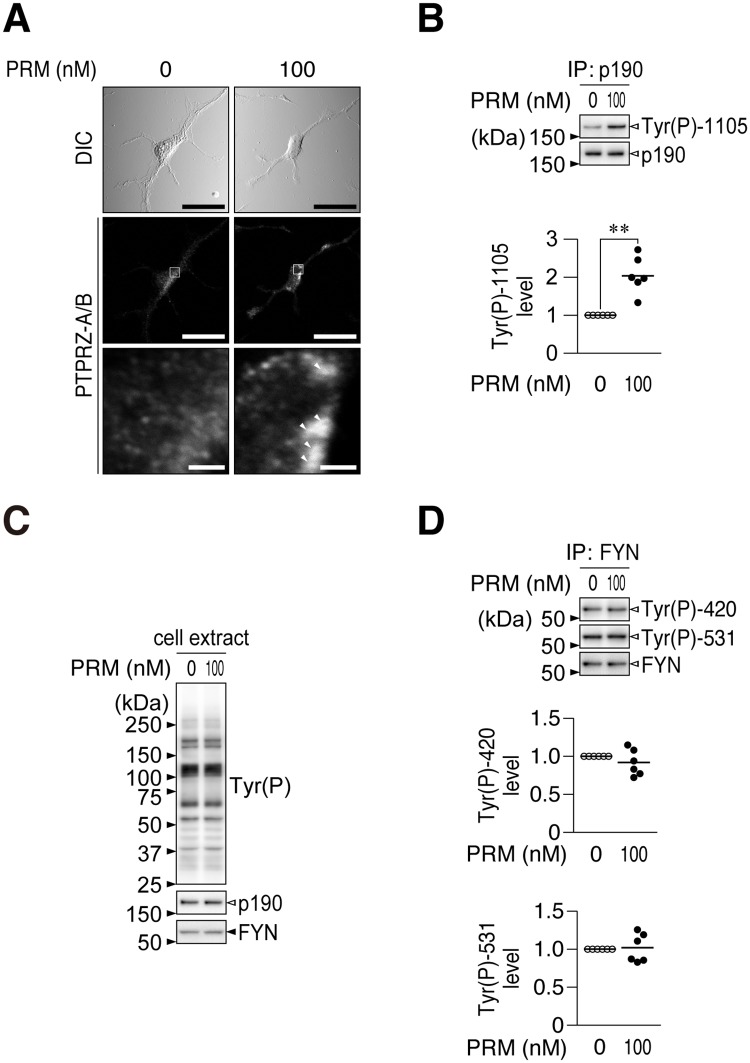
PRM inhibited PTPRZ activity in OL1 cells. (**A**) PTPRZ clustering. OL1 cells treated with PRM or vehicle were fixed with formalin, and then stained with anti-PTPRZ-S. The membrane permeabilization step was omitted to prevent antibody staining of the cytosol, as described previously[41]. The bottom pictures are representative enlarged views of the rectangular region in the middle panels. Arrowheads, anti-PTPRZ-S positive puncta. Scale bars, 100 μm (top and middle) and 10 μm (bottom), respectively. DIC, differential interference contrast. (**B**) Phosphorylation level of p190 RhoGAP at Tyr-1105. OL1 cells cultured on poly-*L*-ornithine-coated dishes were treated with PRM (100 nM) or vehicle (0 nM) for 1 hr. p190 RhoGAP proteins immunoprecipitated (IP) from cell extracts were analyzed by Western blotting using anti-phospho-Tyr-1105 and anti-p190, respectively. The plots show arbitrary densitometric units of phosphorylation levels relative to the vehicle control (six independent experiments). **, *p* < 0.01, significant difference from the vehicle control (Student’s *t*-test). (**C**) Overall tyrosine phosphorylation pattern and protein expression of p190 RhoGAP and FYN. Extracts were analyzed with anti-phosphotyrosine PY20, anti-p190 RhoGAP, and anti-FYN. (**D**) Phosphorylation levels of FYN at Tyr-420 and Tyr-531. FYN proteins were immunoprecipitated and analyzed using anti-Tyr(P)-420, anti-Tyr(P)-531, and anti-FYN, respectively. Full-length blots are presented in S9 Fig.

We then examined the activity of PRM as a ligand mimic for PTPRZ. Immunofluorescent staining of PTPRZ showed that PRM induced the punctate localization of PTPRZ in OL1 cells (Fig 5A). Western blotting indicated that the PRM treatment increased the phosphorylation of p190 RhoGAP at Tyr-1105, which is a dephosphorylation site in a substrate by PTPRZ [43, 44] (Fig 5B). No significant changes were observed in the overall tyrosine phosphorylation patterns of cellular proteins (Fig 5C), or phosphorylation levels of FYN tyrosine kinase (Fig 5D), which phosphorylates p190 RhoGAP [45]. These results indicated that PRM serves as an inhibitory ligand for PTPRZ, similar to pleiotrophin.

We compared the activities of PRM and pleiotrophin in OL1 cell differentiation. As a positive control agent, we also tested miconazole, which reportedly promotes OPC differentiation in cell cultures and remyelination in mouse demyelinating models [30]; however, its molecular target is currently unknown. Although pleiotrophin and miconazole both dose-dependently enhanced the TH-induced differentiation of OL1 cells (Fig 3B and 3C), this elevation was significantly attenuated under aggrecan-coated conditions and markedly smaller than that of PRM (Fig 3D).

**Fig 6:**
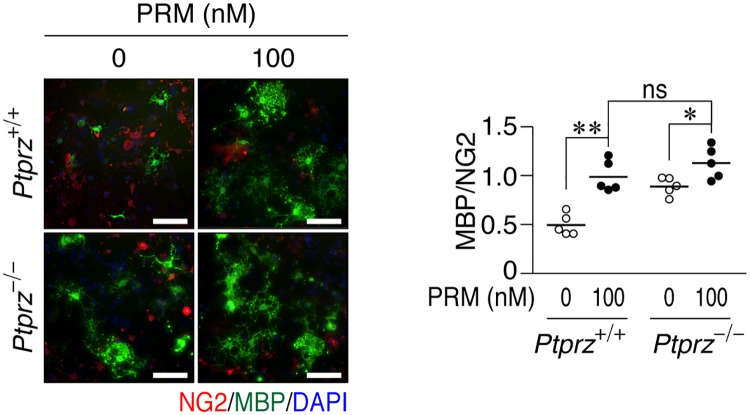
PRM enhanced OPC differentiation in primary cultures. Glial cells prepared from *Ptprz*^+/+^ and *Ptprz*^-/-^ brains were seeded on poly-*L*-ornithine-coated dishes, and cultured for 6 days in differentiation medium without or with 100 nM PRM. Fixed cells were stained with anti-NG2 and anti-MBP in conjunction with DAPI. Scale bars, 100 μm. The plot on the right side shows the ratio of MBP to NG2 (five independent cell culture). *, *p* < 0.05 and **, *p* < 0.01, significant difference between the indicated groups (Student’s *t*-test).

PRM enhanced OPC differentiation in the primary mixed glial culture from wild-type (*Ptprz*^+/+^) mouse brains (Fig 6) as well as that of OL1 cells. Because CSPGs are secreted from astrocytes [35] and cover the substrate, the aggrecan coating was omitted in this primary culture. *Ptprz*-deficient (*Ptprz*^-/-^) glial cells, which are no longer sensitive to pleiotrophin [37], showed higher differentiation levels than wild-type cells (Fig 6). However, in contrast to pleiotrophin, *Ptprz*-deficient and wild-type glial cells both reached a similar level of differentiation by PRM (Fig 6). These results suggest that PRM commonly binds to and neutralizes inhibitory CSPGs including PTPRZ.

### PRM enhanced myelination in the postnatal mouse brain

**Fig 7:**
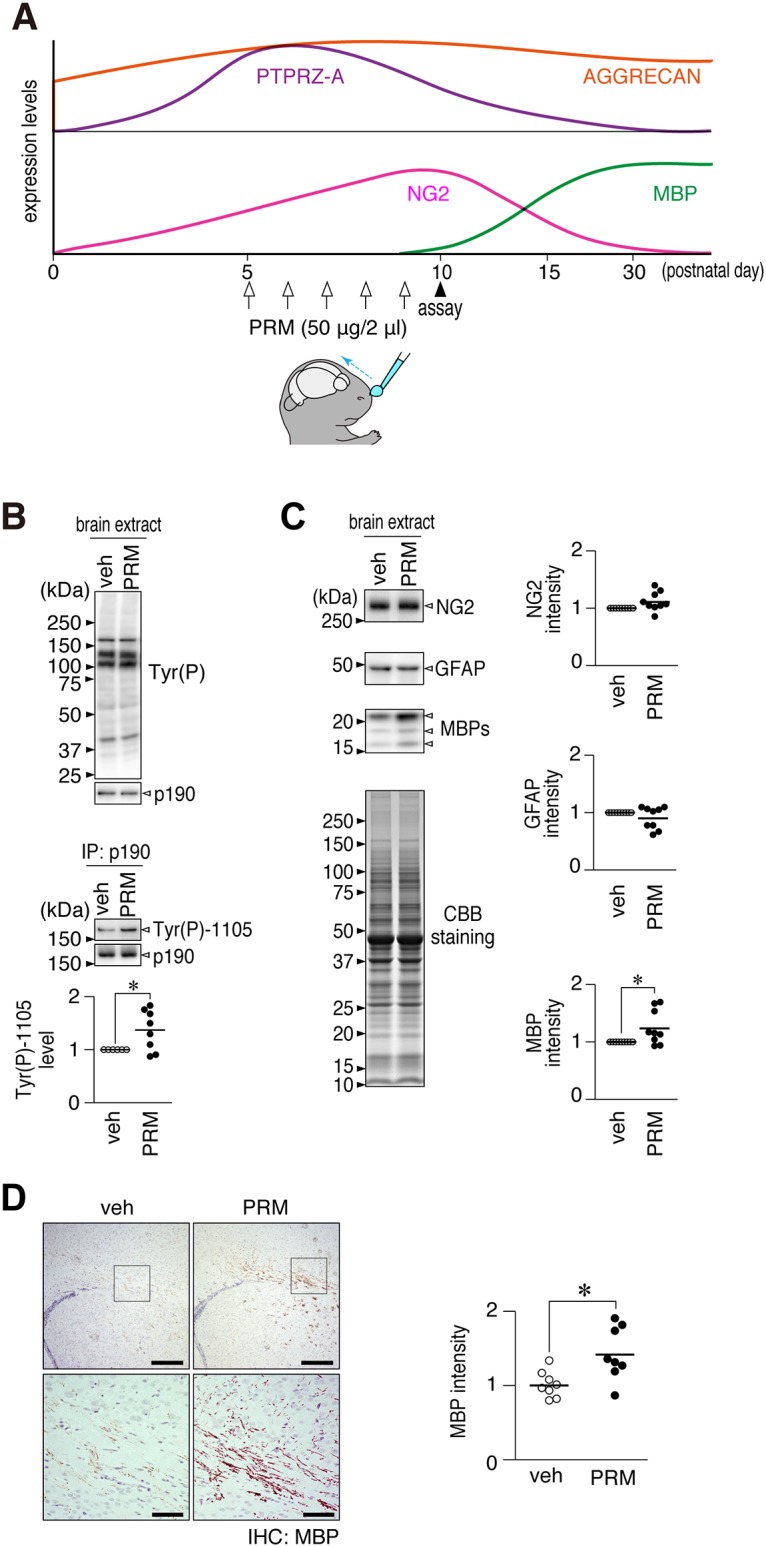
PRM accelerated myelination *in vivo* following its transnasal administration. (**A**) Schematic drawings of the developmental expression patterns of aggrecan [48], PTPRZ-A, NG2, and MBP in the mouse brain [41], and time schedule of the PRM treatment. Mouse pups were treated with PRM (50 μg per day) daily by its transnasal administration from postnatal day 5 (P5) to P9, and sacrificed on P10. (**B**) Overall tyrosine phosphorylation pattern, protein expression of p190 RhoGAP, and Tyr-1105 phosphorylation of p190 RhoGAP in the cerebral cortices. The plot shows the arbitrary densitometric units of Tyr-1105 phosphorylation levels relative to the vehicle control (veh). *, *p* < 0.05, significant difference from the vehicle control (Student’s *t*-test). (**C**) NG2, GFAP, and MBP expression in the cerebral cortices, in which the protein amounts applied were verified by coomassie brilliant blue (CBB) staining. The plots show the arbitrary densitometric units of each protein. *, *p* < 0.05, significant difference from the vehicle control (Student’s *t*-test). (**D**) Immunohistochemical staining of MBP in the mouse brain. The lower pictures are enlarged views of the rectangular region in the upper panels. Scale bars, 200 μm (upper pictures) and 50 μm (lower pictures). The plot on the right side shows the arbitrary densitometric units of the intensity of MBP staining in the corpus callosum. *, *p* < 0.05, significant difference from the vehicle control (Student’s *t*-test). Full-length blots and gels of B and C are presented in S10 Fig.

In the mouse brain, the expression of PTPRZ-A, the major PTPRZ isoform, peaks between postnatal day 5 and 10, and functions to block OPC differentiation and myelination [41]. Since the carboxyl-terminal peptide (VSRRRRRRGGRRRR) of PRM (LMWP) is a cell-penetrating peptide (CPP) [46], which was utilized to facilitate nose-to-brain transport [47], it was expected that PRM itself may promote myelination in the normal developing mouse brain by its intranasal administration. We treated mouse pups daily with PRM or vehicle from postnatal day 5 (see Fig 7A). The tyrosine phosphorylation level of p190 RhoGAP, a PTPRZ substrate, was significantly higher in PRM-administered mouse brains at day 10 than in vehicle-treated pups (Fig 7B). In addition, the expression of MBP, a mature oligodendrocyte marker, was significantly increased, while no changes were observed in the expression of NG2 proteoglycan (an OPC marker) or glial fibrillary acidic protein (GFAP, a reactive astrocyte marker) (Fig 7C). The immunohistochemical staining of MBP also showed a significant increase in MBP-positive myelinated fibers in the corpus callosum upon the administration of PRM (Fig 7D). These results indicated that intranasal administration of PRM is an effective way to stimulate OPC differentiation in the normal developing mouse brain.

### PRM accelerated remyelination after cuprizone-induced demyelination

Cuprizone feeding for 6 weeks induced a similar degree of demyelination between wild-type and *Ptprz*-deficient mice (S4 Fig); however, reparative remyelination was found to be more evident 1–3 weeks after the removal of cuprizone in *Ptprz*-deficient mice than in wild-type mice [37]. The immunostaining intensity of aggrecan was significantly increased in the corpus callosum of wild-type and *Ptprz*-deficient mice after 6 weeks of cuprizone feeding (S4 Fig), indicating that the cuprizone model is useful for examining whether PRM overcomes CSPGs in demyelinating areas *in vivo*. On the other hand, an anti-PTPRZ antibody showed a coarser staining pattern in the corpus callosum with cuprizone feeding, whereas the mean staining intensity did not significantly differ statistically (S5 Fig). The expression of PTPRZ was previously shown to be increased by cuprizone feeding [49, 50]; therefore, we also examined the mRNA expression of *Ptprz*. A quantitative PCR analysis of paraffin sections of the cortex including the corpus callosum showed that its mRNA expression was significantly increased (S5 Fig). This result suggests that the turnover rate of PTPRZ proteins is increased in damaged areas.

**Fig 8:**
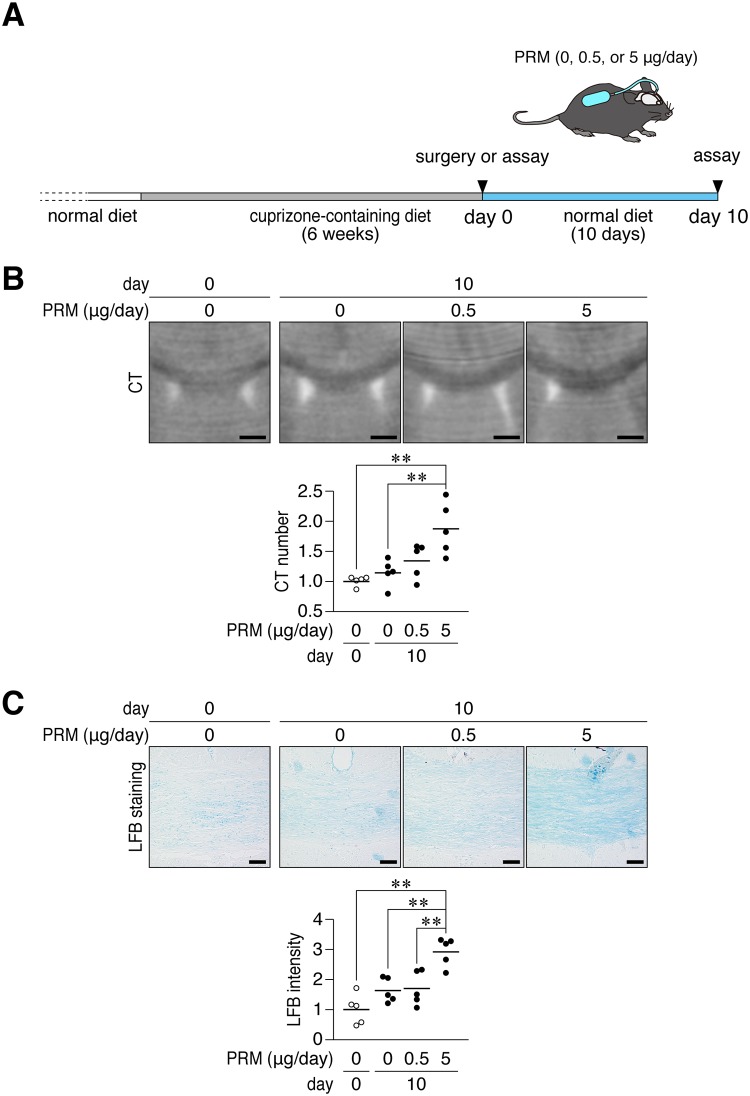
PRM enhanced remyelination after cuprizone-induced demyelination. (**A**) Schematic drawing of cuprizone-induced demyelination and the administration of PRM. Mice were fed a cuprizone-containing diet for 6 weeks. At the end of this period, mice were implanted with osmotic pumps for the intracerebroventricular administration of PRM at 0.5 or 5 μg per day or vehicle for 10 days. (**B**) Coronal plane reconstruction of micro-CT scans. Scale bars, 1 mm. The plots show the relative value of their CT numbers in the dorsal corpus callosum, which was normalized to the value in the non-recovered control. **, *p* < 0.01, significant difference between the indicated groups (analysis of variance with Bonferroni’s *post-hoc* tests). (**C**) LFB staining. Scale bars, 50 μm. The plots show the staining intensity of LFB in the dorsal corpus callosum normalized to the value in the non-recovered control. **, *p* < 0.01, significant difference between the indicated groups (analysis of variance with Bonferroni’s *post-hoc* tests).

We investigated whether PRM stimulates remyelination in adult mice in a cuprizone-induced demyelination model. Following the 6-weeks challenge with cuprizone, we intranasally administered PRM for 10 days; however, no significant recovery effects were observed (S6 Fig). Therefore, we next performed PRM administration using an implanted osmotic pump, through which PRM was continuously delivered (5 μg/day) into the cerebral ventricle for 10 days (Fig 8A). CT imaging of Histodenz-immersed brains [37] showed that recovery from demyelination in the corpus callosum was significantly faster in mice treated with PRM than in the vehicle-treated control (Fig 8B). Luxol fast blue (LFB) myelin staining of tissue sections prepared from the same brains gave similar results (Fig 8C). This recovery was associated with a slight reduction in the number of injured axons, which were positive for the amyloid-β precursor protein (APP) (S7 Fig). On the other hand, the PRM treatment did not affect the distribution of OLIG2-positive oligodendrocyte-lineage cells or accumulation of GFAP-positive reactive astrocytes (S7 Fig).

## Discussion

CSPGs are major components of the extracellular matrix (ECM) in the CNS, but aberrantly accumulated in demyelinating plaques in MS patients [16]. CSPGs are considered to inhibit remyelination by impairing OPC recruitment, oligodendrocyte differentiation, and myelination [15], as well as activate microglia in the inflammatory response at lesion sites [14, 16]. Since undifferentiated OPCs are still present at demyelinating regions, even at the progressive stage of MS, the discovery of compounds capable of boosting the differentiation of OPCs is anticipated. PRM is a polycationic peptide that is widely used clinically to stop the anticoagulant effects of heparin or delay the absorption of insulin and, thus, prolong its effects [40]. To the best of our knowledge, the present study is the first to show that PRM effectively enhances oligodendrocyte differentiation *in vitro* and *in vivo* by acting as a ligand mimic of PTPRZ and neutralizing inhibitory CSPGs.

Several approaches to reduce the levels of CSPGs accumulating in lesioned sites have been reported as novel therapies to enhance the reparation of myelin; enzymatic clearance using chondroitinase ABC [21] and metalloproteinases [51], or the inhibition of CSPG biosynthesis using small molecule inhibitors [15, 35]. However, compounds that fully neutralize the inhibitory activities of CSPGs on oligodendrocyte differentiation have yet to be identified. In the present study, we searched for these agents in the presence of aggrecan using a pure population of premature oligodendrocyte cells (OL1) [37] (Fig 1). We found that PRM fully neutralized the inhibitory activity of aggrecan on OPC differentiation (Fig 3A and 3D). PRM is a commonly used heparin antagonist [40]; however, its antagonistic effects on CSPGs have not yet been demonstrated.

PTPRZ is a membrane-spanning CSPG molecule that is predominantly expressed in OPCs in order to suppress their differentiation through its PTP activity [37, 41, 44]. Negatively charged CS moieties retain PTPRZ in active monomers at the cell membrane, while constituting the high-affinity binding site for endogenous ligands, including pleiotrophin and midkine [42, 52]. The two compose a distinct family of heparin-binding growth factors. Upon the binding of ligand molecules to CS chains via their positively charged domains, PTPRZ molecules begin to cluster and their intracellular PTPases are inactivated [41]. In the present study, PRM mimicked the endogenous ligand for PTPRZ and released the blockage of oligodendrocyte differentiation (Fig 5). Accordingly, PRM accelerated MBP expression in pups (Fig 7), and remyelination in cuprizone intoxication in adult mice (Fig 8). Here, PRM might reduce injury additionally through modulation of the activity of microglia/macrophages *in vivo*, since CSPGs reportedly affect the inflammatory response [14, 16]. As it is difficult by a single mouse model of MS to figure out the effect of PRM on clinical and pathological features of MS [53]. Additional studies are needed to test its potential using other models, such as experimental autoimmune encephalomyelitis (EAE) and lysolecithin-induced demyelination.

Midkine [54] and pleiotrophin [55] partially overcome the inhibition of neurite growth by CSPGs, and improve axon regeneration in injury models of the spinal cord. Similar to its effects on neurons, pleiotrophin improved oligodendrocyte differentiation on aggrecan-coated substrates (Fig 3B), while its activity was markedly lower than that of PRM at the same concentrations (Fig 3D). This result indicates that the masking effects of CSPGs by PRM surpass that by pleiotrophin. The original biological role of PRM is chromatin condensation in sperm [56], in which PRM serves to condense DNA into a compact toroid structure. Aggrecan is a cylindrical-shaped stiff molecule, and the negative charges of CS chains create environments that result in a high osmotic swelling pressure [57]. Glycosaminoglycan (GAG) chains may attenuate their bending rigidity by PRM through charge neutralization and a decrease in the repulsive surface. Further studies are crucial for understanding the molecular basis of the strong neutralizing properties of PRM.

A key obstacle for developing effective drugs for neurological diseases is the blockage of drug entrance into the CNS due to the blood-brain barrier (BBB). Intranasal drug delivery has several advantages, such as bypassing the BBB, minimizing potential side effects in the peripheral system, and non-invasiveness, over direct injections into the brain. Diffuse white matter injury (DWMI), a rather common finding in preterm infants, results in chronic neurodevelopmental disabilities characterized by reduced oligodendrocyte formation due to hypoxia [7]. Recently, intranasal administration of heparin-binding EGF in neonatal mice decreases oligodendroglia death, enhances generation of new oligodendrocytes from progenitor cells, and promotes functional recovery [58], suggesting intranasal treatment is a plausible route to introduce peptides or growth factors into the brain.

LMWP derived from PRM facilitates nose-to-brain transport, and its protein conjugates have the ability to effectively penetrate the brain following their intranasal administration [47]. We herein demonstrated that the intranasal administration of PRM enhanced oligodendrocyte differentiation in the developing mouse brain (Fig 7), though electron microscopy is needed to verify the final myelination. The timing of axonal myelination is critical for the normal development of the CNS, and delayed myelination has been implicated in neurological disorders in young children [5, 6]. Our results may provide an experimental basis for potential approaches to treat delayed myelination.

PRM sulfate is clinically used to reverse the activity of heparin; however, its administration via vein is sometimes associated with several side effects such as systemic hypotension, catastrophic pulmonary vasoconstriction, or allergic reactions [59]. Also, PRM treatment through the intranasal route was not so effective at promoting remyelination of cuprizone-induced lesions in adult mice (S6 Fig) as new-born mice: This may be due to the limited intranasal delivery of PRM in adults as compared to infants. Nonetheless, our findings in the present study suggest that PRM (or its analogues) should be considered as potential remyelinating agents in the future work.

## Materials and methods

### Reagents and antibodies

Aggrecan from bovine articular cartilage (cat no. A1960), and poly-*L*-ornithine hydrobromide (Mw 30,000–70,000; cat no. P3655) were purchased from Sigma-Aldrich. Recombinant human pleiotrophin produced in yeast was described previously [37, 41, 60, 61]. Anti-PTPRZ-S, rabbit polyclonal antibodies against the extracellular region of PTPRZ [62], and rabbit polyclonal antibodies against phosphorylated Tyr-1105 of p190 RhoGAP [43] were described previously. The following are the sources of commercially available regents and antibodies used in the present study: Anti-aggrecan (clone Cat-315; cat no. MAB1581, Millipore), anti-MBP (cat no. sc-13914, Santa Cruz Biotechnology), anti-NG2 proteoglycan (cat no. AB5320, Millipore), anti-phosphotyrosine (clone PY20; cat no. ab16389, Abcam), anti-p190 RhoGAP (cat no. 610150, BD Biosciences; and cat no. 12164, Cell Signaling Technology), anti-FYN (cat no. P2992, Sigma-Aldrich; and cat no. 4023, Cell Signaling Technology), anti-phosphorylated Tyr-416 of Src (cat no. 2101, Cell Signaling Technology), anti-phosphorylated Tyr-527 of Src (cat no. 2105, Cell Signaling Technology), anti-GFAP (cat no. Z0334, Dako), anti-OLIG2 (cat no. AB9610, Millipore), and anti-APP (cat no. ab15272, Abcam).

### PRM and fluorescein-labeled PRM

A commercial product of PRM sulfate salt from salmon was purchased from Sigma-Aldrich (lot no. SLBL0960V, cat no. P4020). We purified PRM using semi-preparative reverse-phase HPLC on a Chemcosorb 7C18 column (10 × 300 mm, Chemco) with a linear gradient of 0–100% acetonitrile containing 0.1% trifluoroacetic acid. The purified sample was pooled and freeze-dried for storage until used. PRM was labeled with fluorescein using 5-(and 6-) carboxyfluorescein succinimidyl ester (NHS-fluorescein, cat no. 46410, Termo Fisher Scientific) according to the manufacturer’s instructions. Briefly, 30 mg PRM sulfate salt was mixed with 3 mg NHS-fluorescein and incubated for 1 hr. Fluorescein-labeled PRM was purified by HPLC, as described above.

### PRM affinity resins

PRM affinity resins were prepared by coupling purified PRM (4 mg) to a HiTrap NHS-activated HP column (1 ml; GE Healthcare) according to the manufacturer’s instructions. Control affinity resins were coupled with BSA (4 mg).

### Ethics statement and experimental animals

All procedures in the present study were approved by the Institutional Animal Care and Use Committee of the National Institutes of Natural Sciences, Japan; approval numbers are 15A096, 16A145, 16A148, and 17A020, and were performed in accordance with the guidelines of the Institutional Committee for the Use of Animals for Research. *Ptprz*-deficient mice [63] were backcrossed with the inbred C57BL/6 strain for more than ten generations. After the final backcross, *Ptprz*^+/-^ mice were interbred to give littermates for analyses. *Ptprz*^+/+^ and *Ptprz*^-/-^ mice were housed under a constant room temperature (23°C) and 50–55% humidity in specific pathogen-free (SPF) conditions on a 8:00 to 20:00 light cycle. All sex-matched littermates were housed in plastic cages (cage size: 12 × 21 × 12.5 cm) with paper-chip bedding (Carfeeaz, Hamri) and food and water were provided *ad libitum*. Surgeries for paraformaldehyde fixation, and implanting osmotic minipumps were performed under isoflurane anesthesia and all efforts were made to minimize suffering.

### OL1 cell culture

Culture dishes (cat no. 353075, CORNING) were coated with the indicated concentrations of poly-*L*-ornithine and aggrecan in 0.2 M NaHCO_3_ and 0.5 M NaCl, pH8.3. OL1 cells [37] were seeded at a density of 7.0 × 10^3^ per well on 96-well plates in serum-free medium containing knockout DMEM/F12 (cat no. 12660, Life Technologies), supplemented with 1× GlutaMAX (cat no. 35050, Life Technologies), 1× StemPro Neural Supplement (cat no. A1050801, Life Technologies), 10 μg/ml of platelet-derived growth factor (PDGF-AA; cat no. 163–19731, Wako Pure Chemical), and 30 ng/ml thyronine and thyroxine (cat no. T2752 and T2376, Sigma-Aldrich). We hereafter called this medium “differentiation medium”. When cells were cultured under “undifferentiated conditions”, thyronine and thyroxine were omitted from the medium. After an overnight culture, the compounds tested were added to the culture. Cells were cultured under a humidified incubator at 37°C with 5% CO_2_.

### Primary mixed glial culture

A primary mixed glial cell culture was performed as described previously [44]. Cortex tissues obtained from mouse brains on postnatal day 1 were dissociated with papain (cat no. LK003150, Worthington Biochemical), and the cells thus isolated were seeded on poly-*L*-ornithine-coated dishes at a density of 5.0 × 10^4^ per 35-mm dish. Cells were cultured in differentiation medium containing DMEM mixed 1:1 with Ham’s F-12 (DMEM/F-12; cat no. 21331, Life Technologies), supplemented with 1× GlutaMAX, 1× N2 supplement (cat no. 17502001, Life Technologies), 0.5% fetal bovine serum (FBS; cat no. 172012, Nichirei Biosciences), 100 μg/ml of bovine serum albumin (BSA; cat no. A4503, Sigma-Aldrich), and 30 ng/ml thyronine/thyroxine. Cells were cultured under a humidified incubator at 37°C with 5% CO_2_.

### Immunocytofluorescence staining

The staining of NG2 and MBP was performed as described previously [44]. Briefly, a fixative containing 4% paraformaldehyde and 20% sucrose in PBS was carefully added to dishes in order to prevent the detachment of cells, and fixation was allowed to proceed for 30 min. After permeabilizing and blocking, cells were incubated with the primary antibodies and then detected with Alexa Fluor-conjugated secondary antibodies. Digital photomicrographs of individual specimens were taken with Biozero BZ-8000 (Keyence) or LSM 700 conforcal microscopy (Zeiss).

### Immunoprecipitation and Western blotting

Cultured cells or mouse brains were extracted with 1% Nonidet P-40 in TBS containing 1 mM vanadate, 10 mM NaF, and protease inhibitors (EDTA-free complete, Roche Molecular Biochemicals). After precleaning the extracts with Protein G Sepharose (cat no. 17–0618, GE Healthcare), the extracts were subjected to immunoprecipitation with a combination of Protein G Sepharose beads and anti-p190 RhoGAP or anti-FYN. The immunocomplexes thus obtained were separated by SDS-PAGE, followed by semi-dry electroblotting onto a polyvinylidene difluoride membrane. After blocking with 4% non-fat dry milk and 0.1% Triton X-100 in TBS, membranes were incubated overnight with the respective antibodies. In order to detect phosphorylated proteins, 1% BSA and 0.1% Triton X-100 in TBS were used for blocking and antibody dilution. The binding of these antibodies was visualized with a chemiluminescent substrate (Luminata forte western HRP substrate, Millipore), and detected using a CCD video camera system (Ez-capture MG, ATTO Bioscience & Technology).

### Transnasal and intracerebroventricular administration

Transnasal administration was performed according to the technique described by Hanson *et al*. [64]. Briefly, mice were held with their necks parallel to the floor, and 2 μl of liquid (50 μg of PRM in saline or vehicle saline) was administrated using a disposable pipette tip (Diamond D10, Gilson).

Intracerebroventricular administration was performed using a micro-osmotic pump (infusion rate, 0.5 μl per hour; model no. 1007, Alzet) according to the manufacturer’s instructions. Briefly, a pre-cut 15-mm-long cannula (21G, Inter Medical) was attached to the pumps via a flexible ~6-cm-long catheter, and filled with a solution containing either vehicle (saline) or PRM solution (0.42 or 0.042 mg/ml). The cannulas were stereotaxically implanted into the left lateral cerebral ventricle at a depth of 2.5 mm (coordinates with respect to the bregma: 0.22 mm anterior and 1.0 mm lateral). The pump units were subcutaneously implanted. Mice were anesthetized with 2% vaporized isoflurane during the surgery.

### Cuprizone-induced demyelination

Demyelination was induced in 2-month-old male mice by feeding powdered mouse chow (Rodent Diet CA-1, cat no. 0000515, CLEA) containing 0.2% (*w*/*w*) cuprizone (bis-cyclohexanone oxaldihydrazone; cat no. C9012, Sigma-Aldrich) for 6 weeks. Micro-osmotic pumps were implanted as above on the last day of cuprizone feeding, and the diet was returned to the normal pellet diet of CA-1 for the induction of remyelination.

### Microcomputed tomography and histochemistry

Microcomputed tomography (micro-CT) imaging was performed as described previously [37]. In brief, mouse brains fixed with 4% paraformaldehyde were immersed in a graded series of Histodenz [5-(N-2,3-dihydroxypropylacetamido)-2,4,6-triiodo-N,N’-bis (2,3-dihydroxypropyl) isophthalamide; cat no. D2158, Sigma-Aldrich] solutions. Specimens were scanned on the micro-CT system R_mCT2 (Rigaku) at 90 kV (160 μA) with a 20-mm field of view, and data were reconstructed using OsiriX software (Pixmeo).

After CT scanning, the brains were subjected to conventional paraffin embedding, and coronal sections (thickness of 4 μm) were prepared. The LFB staining of sections was performed with a commercial kit (Luxol fast blue stain solution, cat no. 4102, Muto Pure Chemicals). In order to detect PTPRZ, aggrecan, OLIG2, and APP, sections were microwaved in 10 mM citrate buffer (pH 6.0) at 98°C for 5 min as an antigen retrieval step. The bound primary antibodies were visualized with HRP-conjugated secondary antibodies along with the Dako liquid diaminobenzidine chromogen system (Life Technologies). Digital photomicrographs of individual specimens were taken with the Eclipse microscope Ci-L using the DS-Fi2 CCD camera (Nikon).

### Quantitative real-time PCR

Total RNA was isolated from paraffin-embedded tissue sections of mouse brains using Nucleospin totalRNA FFPE (cat no. 740982, Takara Bio) according the manufacturer’s instructions. cDNAs were synthesized using the Prime script RT reagent kit with gDNA Eraser (cat no. RR047, Takara Bio). Real-time PCR was performed using a commercial kit (SYBR Premix Ex Taq II; cat no. RR820, Takara Bio) on a real-time PCR system (StepOnePlus Real Time PCR System, Thermo Fisher). The sequences of the primer set (Perfect real-time primer support system, Takara Bio) are as follows: *Ptprz*; forward, 5’-GAATCCTGCAGAGCTTCCTC-3’ and reverse 5’-TGTCTGTAGTATCCATAAGCCCAGT-3’; and glyceraldehyde-3-phosphate dehydrogenase (*Gapdh*); forward, 5’-ATGGTGAAGGTCGGTGTG-3’ and reverse 5’-GTCGTTGATGGCAACAATC-3’. The relative quantities of target mRNA were normalized to GAPDH.

### Image and statistical analyses

Quantitative image analyses were performed using Adobe Photoshop CS6 software (Adobe Systems). Statistical analyses were performed using IBM SPSS Statistics 20 software.

## Supporting information

S1 Fig PRM binding to aggrecan. (PDF)

S2 Fig PRM alone without thyroid hormones did not induce cell differentiation in OL1 cells. (PDF)

S3 Fig PRM binding to OL1 cells. (PDF)

S4 Fig Aggrecan expression was induced following cuprizone-induced demyelination. (PDF)

S5 Fig PTPRZ expression in cuprizone-lesioned mouse brains. (PDF)

S6 Fig Transnasal administration of PRM to cuprizone-lesioned mice. (PDF)

S7 Fig Histological evaluations of effects of PRM during recovery from cuprizone-induced lesions. (PDF)

S8 Fig Full-length blots for Fig 4. (PDF)

S9 Fig Full-length blots for Fig 5. (PDF)

S10 Fig Full-length blots and gels for Fig 7. (PDF)
