# Supplementary material for: Protamine neutralizes chondroitin sulfate proteoglycan-mediated inhibition of oligodendrocyte differentiation
Source: PLoS One. 2017 Dec 7;12(12):e0189164. doi: 10.1371/journal.pone.0189164 (PMC5720700; doi:10.1371/journal.pone.0189164)
Supplement: S1 Fig — (PDF) [file pone.0189164.s001.pdf]

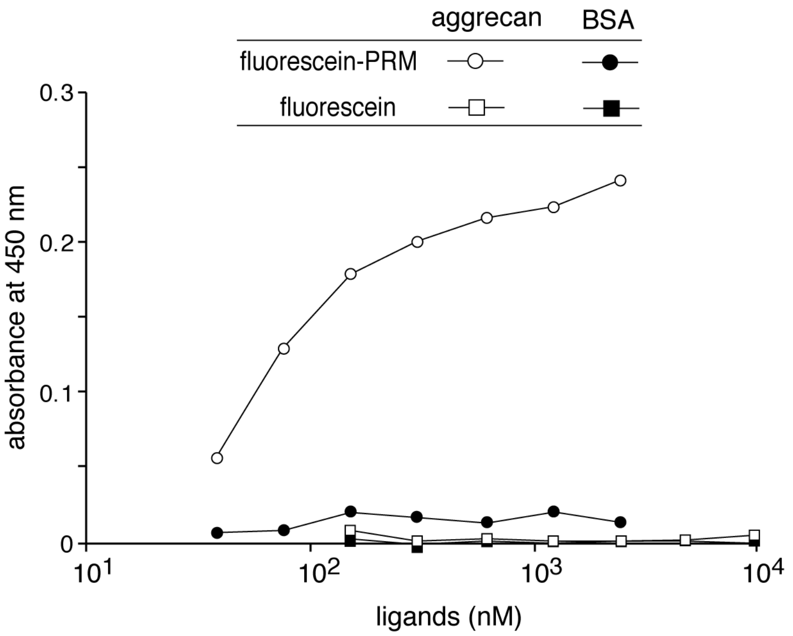

S1 Fig. PRM binding to aggrecan. A 96-well polypropylene microtiter plate was coated overnight with 5 µg/ml aggrecan or BSA in 0.1 M $NaHCO_3$ and 0.5 M NaCl, pH8.5. After blocking with 1% BSA and 0.1% Tween 20 in TBS, a dilution series of fluorescein-labeled PRM or fluorescein in the blocking buffer was added and incubated for 90 min. Horseradish peroxidase (HRP)-conjugated anti-fluorescein (cat no. NEF710001EA, PerkinElmer) was then added and incubated for 90 min. Color development was performed using the SureBlue Reserve TMB Substrate (cat no. 53-00-01, KPL) according to the manufacturer's instructions, and absorbance was measured at 450 nm (SH-9000Lab, Corona Electric). Absorbance values were corrected for the background without fluorescein molecules.
