# Supplementary material for: Protamine neutralizes chondroitin sulfate proteoglycan-mediated inhibition of oligodendrocyte differentiation
Source: PLoS One. 2017 Dec 7;12(12):e0189164. doi: 10.1371/journal.pone.0189164 (PMC5720700; doi:10.1371/journal.pone.0189164)
Supplement: S2 Fig — (PDF) [file pone.0189164.s002.pdf]

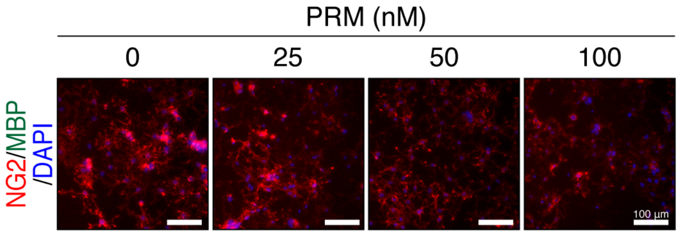

S2 Fig. PRM alone without thyroid hormones did not induce cell differentiation in OL1 cells. Double-immunofluorescence labeling of OL1 cells. OL1 cells were cultured in non-differentiated medium without thyroid hormones on dishes coated with poly-*L*-ornithine for 10 days, and examined as in Figure 3. Scale bars, 100 µm.
