# Supplementary material for: Protamine neutralizes chondroitin sulfate proteoglycan-mediated inhibition of oligodendrocyte differentiation
Source: PLoS One. 2017 Dec 7;12(12):e0189164. doi: 10.1371/journal.pone.0189164 (PMC5720700; doi:10.1371/journal.pone.0189164)
Supplement: S3 Fig — (PDF) [file pone.0189164.s003.pdf]

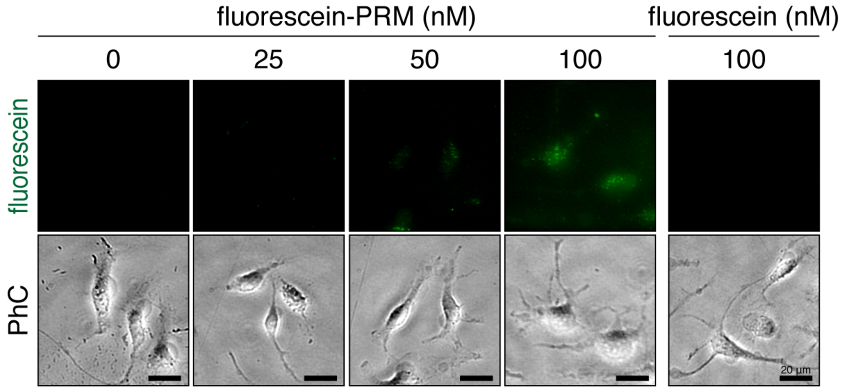

S3 Fig. PRM binding to OL1 cells. OL1 cells cultured on poly-*L*-ornithine-coated plates were incubated with fluorescein-labeled PRM or fluorescein for 1 hr. After washing with PBS, cells were fixed and observed at excitation/emission wavelengths of 518/484 nm using a fluorescence microscope (Biozero BZ-8000, Keyence). Scale bars, 20 µm. Fluorescein-labeled PRM, but not fluorescein itself showed binding to OL1 cells. PhC, Phase contrast.
