# Supplementary material for: Protamine neutralizes chondroitin sulfate proteoglycan-mediated inhibition of oligodendrocyte differentiation
Source: PLoS One. 2017 Dec 7;12(12):e0189164. doi: 10.1371/journal.pone.0189164 (PMC5720700; doi:10.1371/journal.pone.0189164)
Supplement: S4 Fig — (PDF) [file pone.0189164.s004.pdf]

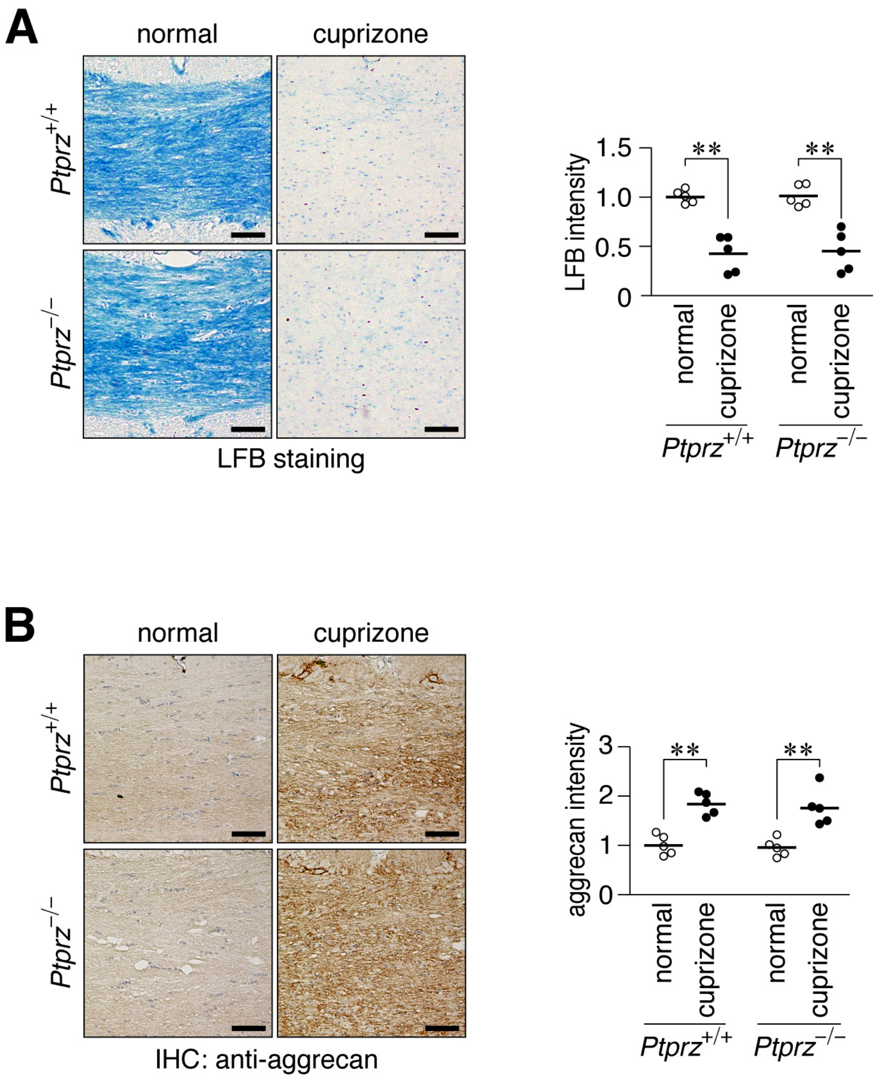

S4 Fig. Aggrecan expression was induced following cuprizone-induced demyelination. (**A**) LFB staining of the dorsal corpus callosum. Wild-type (*Ptprz*$^{+/+}$) and *Ptprz*-deficient (*Ptprz*$^{-/-}$) mice were fed a cuprizone-containing diet for 6 weeks (cuprizone) or were maintained on the regular diet (normal). Scale bars, 50 µm. The plot shows the quantification of staining intensity in the dorsal corpus callosum normalized to the staining intensity in normal *Ptprz*$^{+/+}$ mice. **, $p < 0.01$, significant difference between the indicated groups (analysis of variance with Bonferroni's *post-hoc* tests). (**B**) Anti-aggrecan staining of the sections shown in A. Scale bars, 50 µm. The plot shows the staining intensity in the dorsal corpus callosum normalized to normal *Ptprz*$^{+/+}$ ones. **, $p < 0.01$, significant difference between the indicated groups (analysis of variance with Bonferroni's *post-hoc* tests).
