# Supplementary material for: Protamine neutralizes chondroitin sulfate proteoglycan-mediated inhibition of oligodendrocyte differentiation
Source: PLoS One. 2017 Dec 7;12(12):e0189164. doi: 10.1371/journal.pone.0189164 (PMC5720700; doi:10.1371/journal.pone.0189164)
Supplement: S5 Fig — (PDF) [file pone.0189164.s005.pdf]

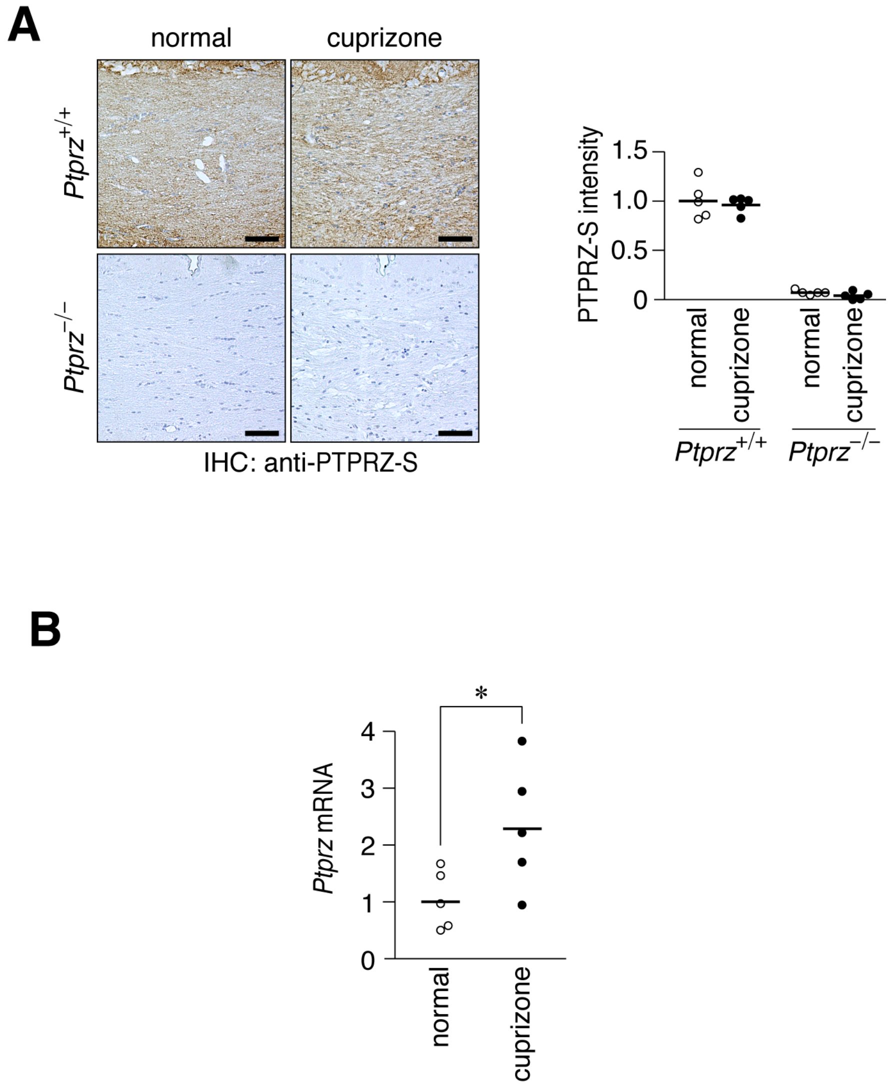

S5 Fig. PTPRZ expression in cuprizone-lesioned mouse brains. (**A**) Anti-PTPRZ-S staining of sections shown in supplementary figure S4. Scale bars, 50 µm. The plot shows the staining intensity in the dorsal corpus callosum normalized to normal *Ptprz*$^{+/+}$ ones. **, $p < 0.01$, significant difference between the indicated groups (analysis of variance with Bonferroni's *post-hoc* tests). Aggrecan immunoreactivity in the corpus callosum. (**B**) Quantitative RT-PCR analyses. Total RNA was extracted from the paraffin-embedded tissue sections of *Ptprz*$^{+/+}$ mice shown in A, and then subjected to quantitative RT-PCR. The plots show the mRNA expression of *Ptprz* as normalized to *Gapdh* expression. *, $p < 0.05$ (Student's *t*-test).
