# Supplementary material for: Protamine neutralizes chondroitin sulfate proteoglycan-mediated inhibition of oligodendrocyte differentiation
Source: PLoS One. 2017 Dec 7;12(12):e0189164. doi: 10.1371/journal.pone.0189164 (PMC5720700; doi:10.1371/journal.pone.0189164)
Supplement: S6 Fig — (PDF) [file pone.0189164.s006.pdf]

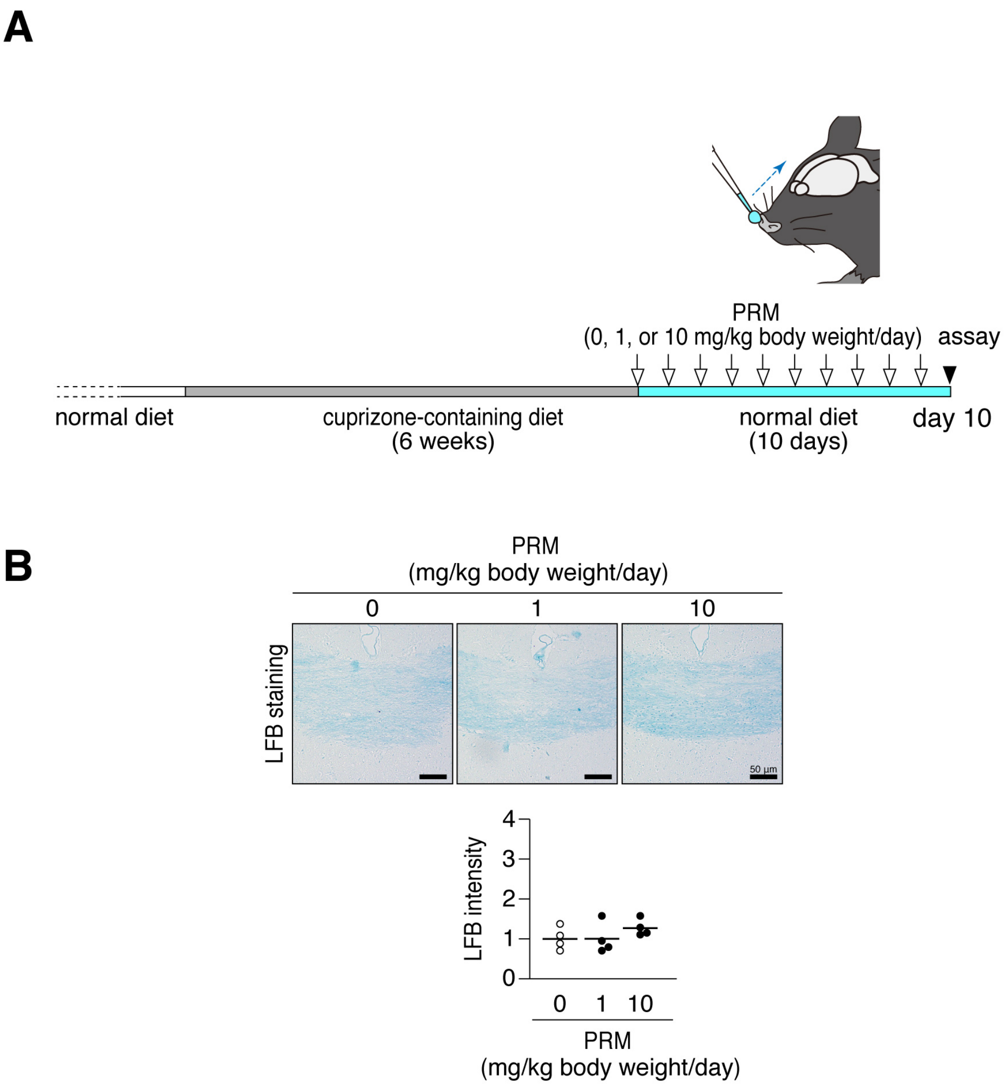

S6 Fig. Transnasal administration of PRM to cuprizone-lesioned mice. (**A**) Schematic drawing representing the treatment schedule. Mice were fed a cuprizone-containing diet for 6 weeks. At the end of the period, mice were treated daily with PRM (0, 1, or 10 mg/kg body weight per day) by its transnasal administration for 10 days. (**B**) LFB staining. The plot shows the intensity of LFB staining in the dorsal corpus callosum. No significant differences were detected by a one-way analysis of variance. Scale bars, 50 µm.
