# Supplementary material for: Protamine neutralizes chondroitin sulfate proteoglycan-mediated inhibition of oligodendrocyte differentiation
Source: PLoS One. 2017 Dec 7;12(12):e0189164. doi: 10.1371/journal.pone.0189164 (PMC5720700; doi:10.1371/journal.pone.0189164)
Supplement: S7 Fig — (PDF) [file pone.0189164.s007.pdf]

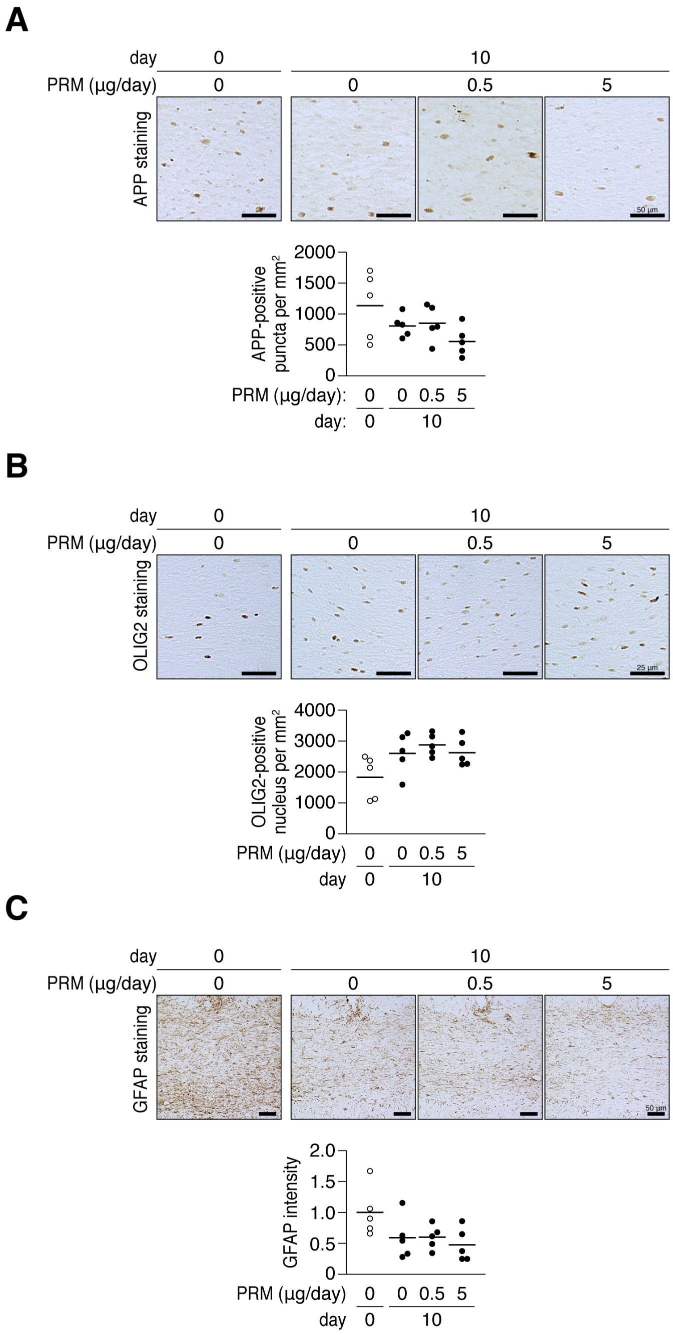

S7 Fig. Histological evaluations of effects of PRM during recovery from cuprizone-induced lesions. (**A-C**) Immunohistochemical analyses of the sections shown in Figure 8C using anti-APP (damaged axons, A), anti-OLIG2 (a marker of oligodendrocyte-lineage cells, B), or anti-GFAP (reactive astrocytes, C). Images show the dorsal corpus callosum. Scale bars, 50 μm (A and C) or 25 μm (B). The lower plots show the number of anti-APP-stained puncta (A), number of anti-OLIG2-stained nuclei (B), or intensity of anti-GFAP staining (C), respectively, in the dorsal corpus callosum. No significant differences were detected by a one-way analysis of variance.
