# Supplementary figures and images for: Protamine neutralizes chondroitin sulfate proteoglycan-mediated inhibition of oligodendrocyte differentiation
Source: PLoS One. 2017 Dec 7;12(12):e0189164. doi: 10.1371/journal.pone.0189164 (PMC5720700; doi:10.1371/journal.pone.0189164)

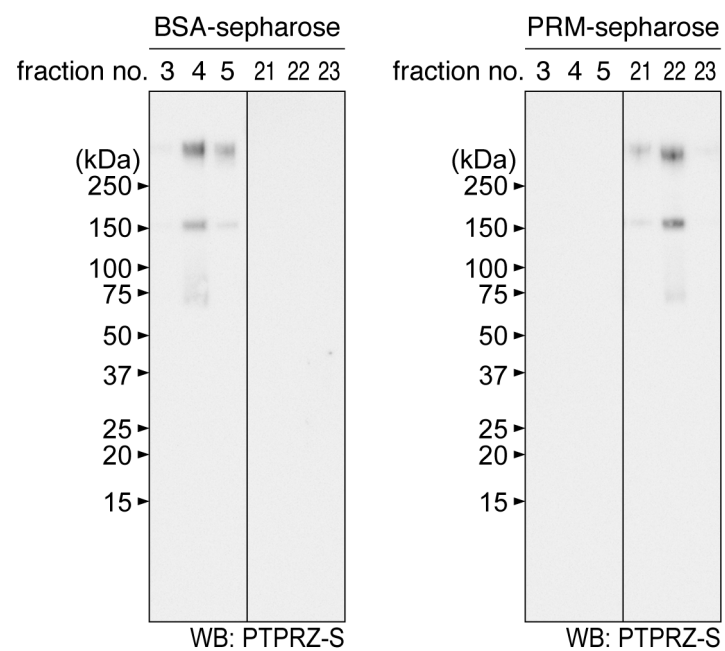

**S8 Fig. Full-length blots for Fig 4.**

Supplement: S8 Fig — (PDF) [file pone.0189164.s008.pdf]

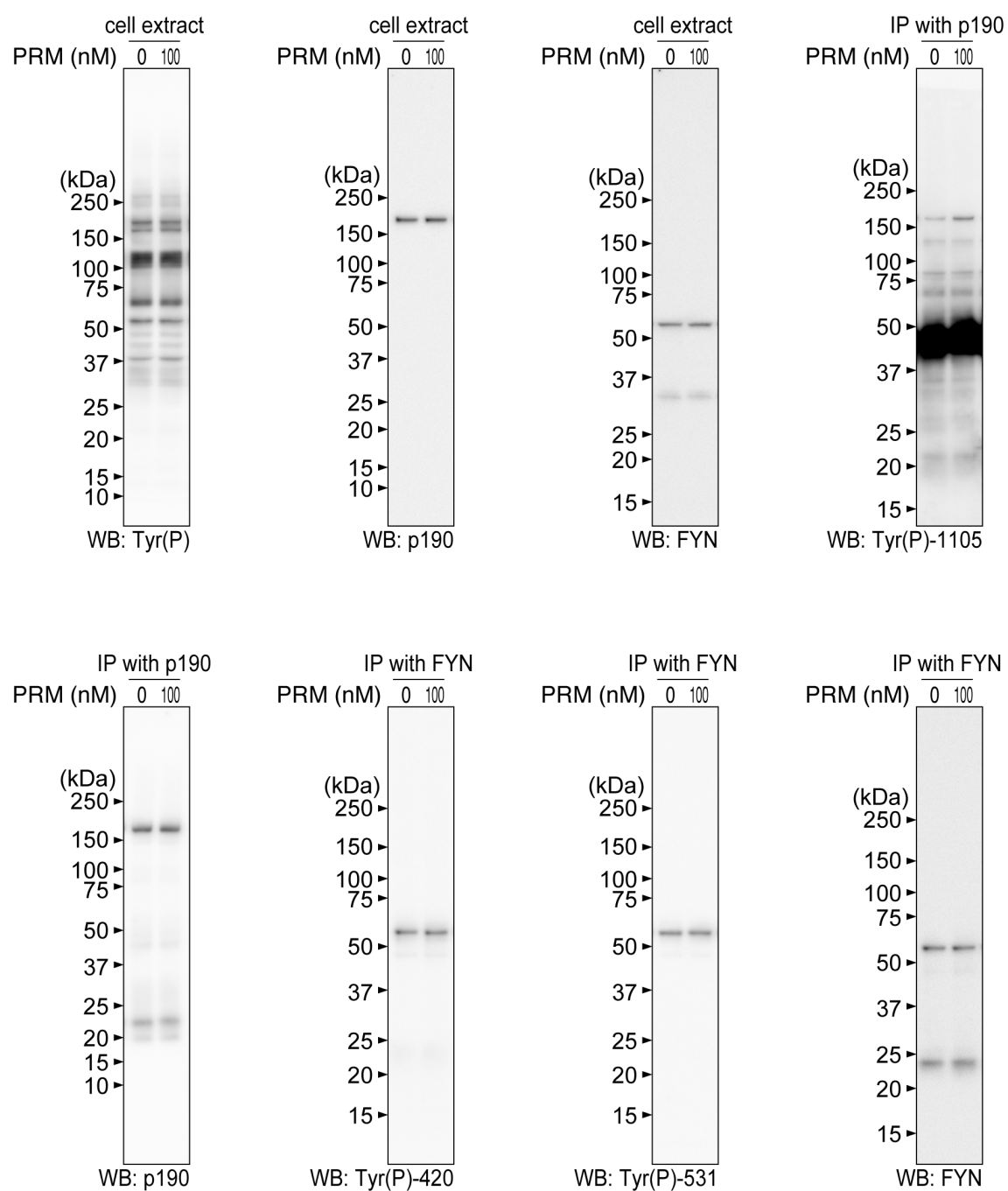

**S9 Fig. Full-length blots for Fig 5.**

Supplement: S9 Fig — (PDF) [file pone.0189164.s009.pdf]

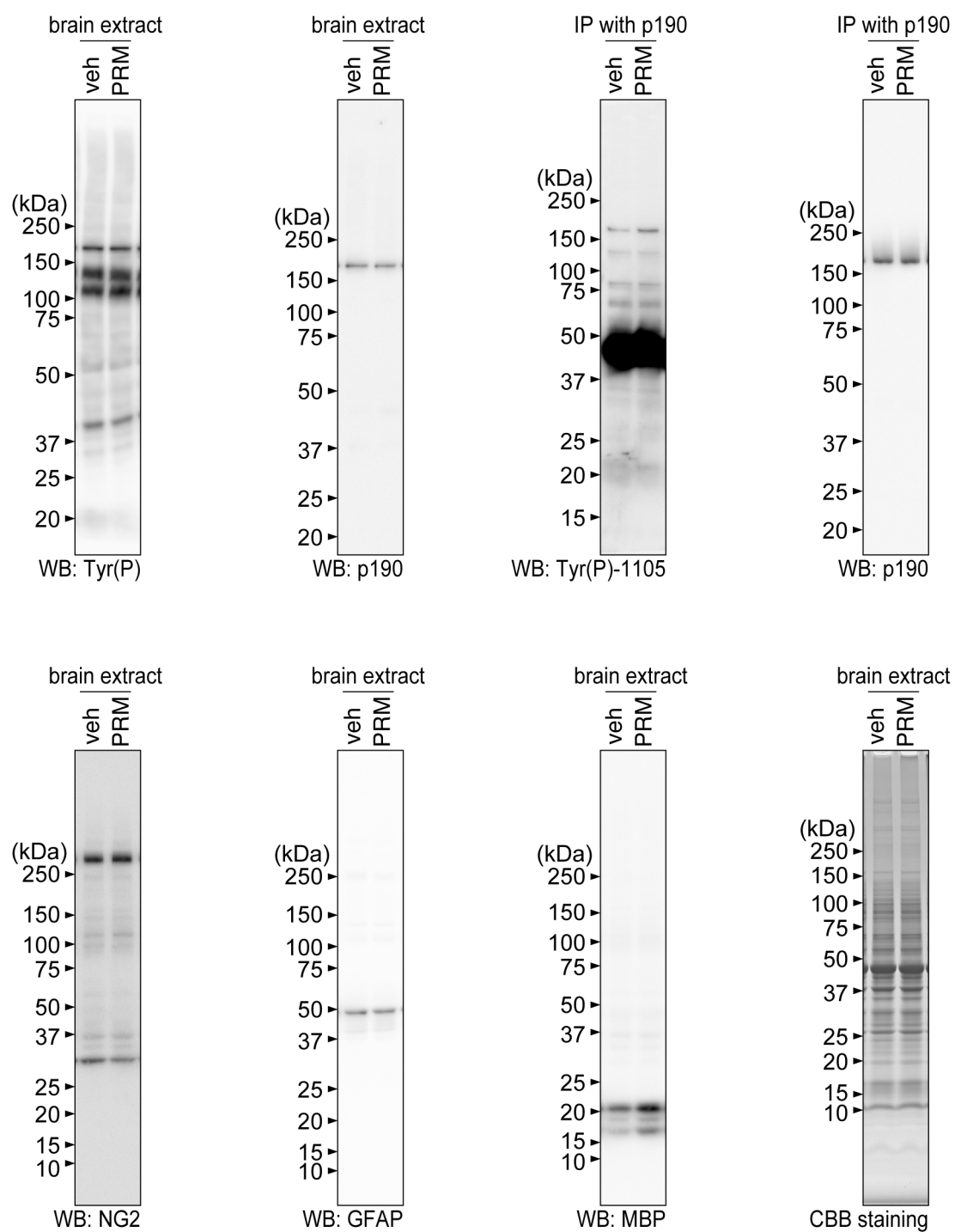

**S10 Fig. Full-length blots and gels for Fig 7.**

Supplement: S10 Fig — (PDF) [file pone.0189164.s010.pdf]
